# Chronic phase advances reduces recognition memory and increases vascular cognitive dementia-like impairments in aged mice

**DOI:** 10.1038/s41598-024-57511-2

**Published:** 2024-04-02

**Authors:** Jennifer A. Liu, Jacob R. Bumgarner, William H. Walker, O. Hecmarie Meléndez-Fernández, James C. Walton, A. Courtney DeVries, Randy J. Nelson

**Affiliations:** 1 Department of Neuroscience, Rockefeller Neuroscience Institute, West Virginia University, Morgantown, USA; 2 Department of Medicine, West Virginia University, Morgantown, USA; 3 West Virginia University Cancer Institute, West Virginia University, Morgantown, USA

**Keywords:** Circadian rhythms and sleep, Risk factors, Cognitive ageing, Spatial memory, Working memory, Alzheimer's disease

## Abstract

Disrupted or atypical light–dark cycles disrupts synchronization of endogenous circadian clocks to the external environment; extensive circadian rhythm desynchrony promotes adverse health outcomes. Previous studies suggest that disrupted circadian rhythms promote neuroinflammation and neuronal damage post-ischemia in otherwise healthy mice, however, few studies to date have evaluated these health risks with aging. Because most strokes occur in aged individuals, we sought to identify whether, in addition to being a risk factor for poor ischemic outcome, circadian rhythm disruption can increase risk for vascular cognitive impairment and dementia (VCID). We hypothesized that repeated 6 h phase advances (chronic jet lag; CJL) for 8 weeks alters cerebrovascular architecture leading to increased cognitive impairments in aged mice. Female CJL mice displayed impaired spatial processing during a spontaneous alternation task and reduced acquisition during auditory-cued associative learning. Male CJL mice displayed impaired retention of the auditory-cued associative learning task 24 h following acquisition. CJL increased vascular tortuosity in the isocortex, associated with increased risk for vascular disease. These results demonstrate that CJL increased sex-specific cognitive impairments coinciding with structural changes to vasculature in the brain. We highlight that CJL may accelerate aged-related functional decline and could be a crucial target against disease progression.

## Introduction

Vascular-related cognitive impairment is the second most common form of dementia, a neurodegenerative disorder broadly characterized by impaired cognition that is attributable to cerebrovascular pathology. This clinical condition comprises diminished executive function, spatial memory, and attention, leading to impaired general cognition^1,2^. Working memory is one of the important components of cognition that is affected by aging and dementia; but despite cognitive impairments being well characterized in foundational and clinical scientific literature, there are few studies that have uncovered pathophysiological mechanisms underlying cognitive decline.

In mammals, circadian rhythms, with periods of ~ 24 h, are generated by the paired suprachiasmatic nuclei (SCN) of the anterior hypothalamus^3^. External cues such as exposure to light synchronize (entrain) the internal clock to the external environment and become set precisely to 24 h. These rhythms are important for synchronizing physiology and behavior to the solar day to optimize homeostatic function and survival. However, disruptions to these rhythms resulting from frequent periods of circadian misalignment including, for example, social jet lag or repeated travel, have been documented to adversely affect health^4–6^. Further, the prevalence of dementia-like impairments and mood disturbances display rhythmic variations across light dark cycles, characterized as “sundowning syndrome”, which highlights the potential involvement of circadian rhythms in this neurodegenerative disease progression^7^.

Additionally, other studies assessing manipulations of the circadian clock and cognitive function report increased risk for cognitive impairments across adult and aged individuals, as well as neurodegenerative and neuropsychiatric disorders^8,9^. Further, individuals exposed to night shift work or other forms of dysregulated circadian rhythms display elevated health risks. For example, changes in rhythmicity of the clock gene basic helix-loop-helix ARNT like 1 (*Bmal1*) have been observed in the early stages of Alzheimer disease (AD) patients^10^. In studies using triple transgenic murine models of AD (3 × Tg-AD), expression of clock genes is disrupted in the SCN and various hypothalamic regions during the early stages of neuropathology and cognitive impairment^11^. Collectively, these studies highlight and emphasize the need for investigation into circadian influences on disease progression and health outcomes, particularly, in aged individuals that are more susceptible to perturbation of physiological function.

Despite an increasing number of studies associating disrupted circadian rhythms with increased health risks, few studies to date have directly evaluated the role of circadian rhythm disruption in aged populations. Further, the pathophysiological mechanisms responsible for vascular influences on dementia remain poorly characterized. Therefore, we test the hypothesis that disrupted circadian rhythms, induced by chronic phase advances (experimental chronic jet lag; CJL), alters vasculature in the brain and produces a vascular cognitive impairment and dementia phenotype in aged mice. If so, then exposure to circadian disruption may be a modifiable risk factor for vascular contributions to cognitive impairment and dementia (VCID). To test this hypothesis, we exposed > 20-month-old mice to a chronic jet lag paradigm, consisting of a 6 h phase advance once every 7 days for 8 consecutive weeks, and then performed two assessments of cognitive function in male and female aged mice. Following assessment, we examined vascular networks and morphology in the brain using 3-dimensional resin casting to provide insight into the state of brain health and disorder in aged mice after chronic circadian rhythm disruption. Our results demonstrate that chronic phase advances result in sex specific cognitive impairments, and that these changes coincide with structural tortuosity changes to vasculature in the brain. These results reflect that disruption to circadian rhythms likely contributes to accelerated age-related decline and greater risk factors for health impairment in aged populations.

## Methods

**Figure 1:**
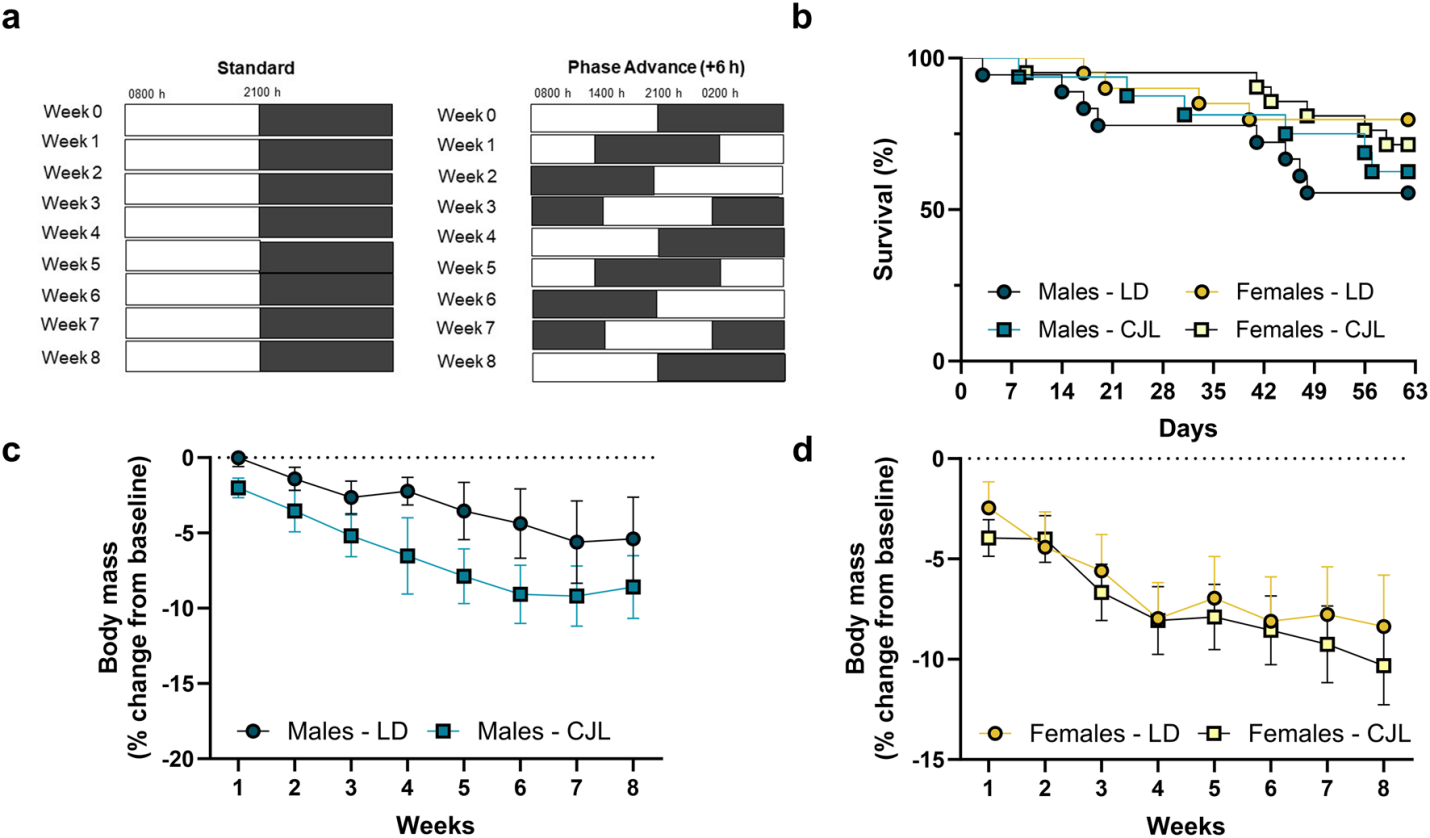
Chronic phase advances do not accelerate body mass loss or increase mortality in aged mice. (**a**) Schematic illustration of experimental design and timeline for stable circadian conditions or chronic phase advances across 8 weeks (**b**) Survival plot of aged male and female C57Bl/6 mice across 8 weeks of stable circadian conditions or chronic jet lag (CJL). Survival does not differ between sex or lighting condition (p > 0.05) (Male LD n = 10/18, Male CJL n = 10/16, Female LD n = 16/20, Female CJL n = 15/21). Body mass across 8 weeks in either stable circadian conditions or CJL did not differ between lighting conditions (p > 0.05) for either (**c**) males or (d) females. Mice that died prior to the experimental endpoint were excluded from body mass analyses. Data are represented as Mean ± SEM (Male LD n = 10, Male CJL n = 10, Female LD n = 16, Female CJL n = 15) *p < 0.05.

**Figure 2:**
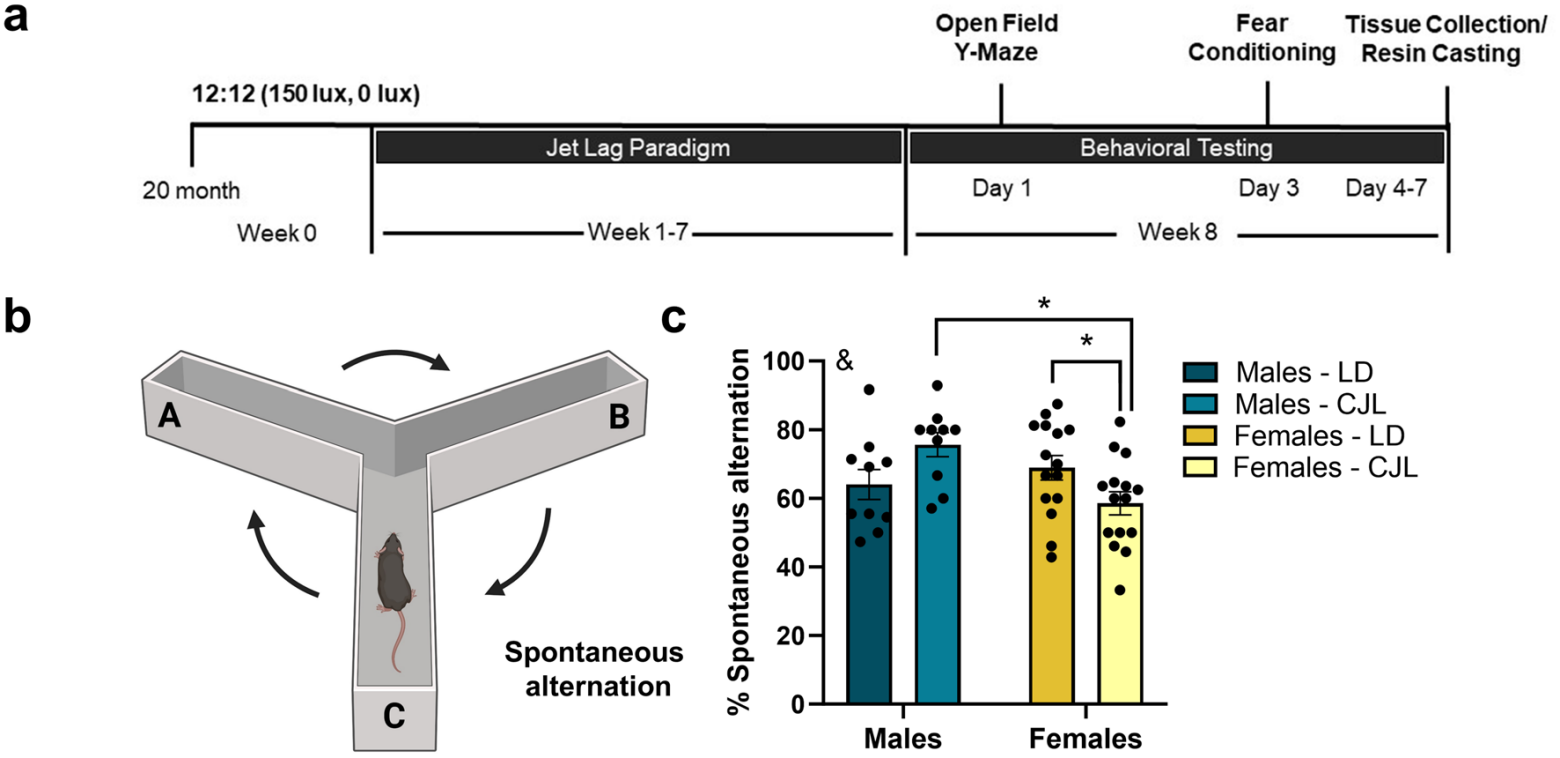
Chronic phase advances reduce spatial working memory in female mice. (**a**) Schematic illustration of experimental design and timeline of experiments and behavioral tests. (**b**) Schematic illustration of Y-maze for spontaneous alternation task. (**c**) Females exposed to chronic phase advances have impaired spatial working memory compared to mice in stable circadian controls, including LD males and LD females, as well as CJL males (p < 0.05). Data are represented as Mean ± SEM (Male LD n = 10, Male CJL n = 10, Female LD n = 15, Female CJL n = 15). & symbol depicts a statistically significant interaction between sex and lighting condition, *p < 0.05. One LD female was excluded from spontaneous alternation tasks due to a corrupted video file.

Seventy-six 16-month-old C57BL/6 male and female mice were obtained from the US National Institute of Aging colony and were housed until 20 months of age prior to experimental group assignment. Mice were single-housed and fed ad libitum access to standard rodent chow (2018 Teklad) and reverse osmosis water. After acclimation, mice were assigned to remain in stable light–dark (LD) cycles (12 h light:12 h dark; lights on 08:00 h EST) or were exposed to a 6 h phase advance in light cycle once each week for eight weeks (Fig. 1a). Body mass was measured weekly, and mice were checked daily during the light phase and survival was recorded. On week 8, behavioral testing was conducted to assess spatial working memory using the spontaneous alternation Y-maze task, and novel object recognition task; open field tests were conducted to assess locomotor activity and anxiety-like behavior, and amygdala dependent cognitive performance was assessed through auditory-cued associative learning and contextual fear conditioning (Fig. 2a). To avoid disrupting circadian rhythms via light exposure from behavioral testing apparati (i.e., auditory-cued associative learning chamber), behavioral testing was conducted during the light phase (zeitgeber time (ZT) 1–8, 09:00 h to 16:00 h) starting at 09:00 h. Mice were tested in two separate cohorts to allow adequate sample sizes for statistical analysis due to a limited number of mice that can be behaviorally tested per day (cohort 1: LD males n = 8, LD females n = 11, CJL males n = 8, CJL females n = 12; cohort 2: LD males n = 10, LD females n = 10, CJL males n = 8, CJL females n = 9). One female LD mouse was removed from the first cohort study and excluded from any analyses due to self-injuries a week after starting the experiment. All studies were approved by the West Virginia University Institutional Animal Care and Use Committee, and animals were maintained in accordance with the NIH Animal Welfare guidelines. Reporting of animal data in this manuscript followed recommendations included in the ARRIVE guidelines.

### Spontaneous alternation task

Spatial working memory was assessed for each mouse during free exploration of a Y-Maze apparatus (35 cm at each arm, converged at an equal angle). Mice were habituated to the testing room 30 min prior to behavioral testing. Following habituation, mice were placed into one arm of the maze and allowed to freely move through the maze for 180 s; total locomotor activity and exploratory behavior (arm entries) were recorded and tracked using Ethovision XT (Noldus). Arm entries were defined as all four limbs crossing into the arm, and an alternation defined as entering 3 consecutive novel arms. Percent alternation, a measure of spatial working memory, was calculated using the following formula: [(# alternations/maximum possible number of alternations) × 100]. Maximum possible alternations were the total arm entries − 2.

### Open field

Locomotor exploratory and anxiety-like behaviors were assessed in a 36 cm × 36 cm polypropylene open field arena housed in a sound- and light-attenuating chamber, equipped with infrared sensors to detect horizontal and vertical movement (Open Field Photobeam Activity System, San Diego Instruments Inc., San Diego, CA USA) as previously described^12^. Mice were habituated to the testing room 30 min prior to behavioral testing. Total locomotor activity across 10 min of free exploration of the novel arena was determined by the total number of beam breaks. Percent central tendency, a measure of exploratory behavior inversely related to anxiety, was calculated as (total # of beam breaks in the central area of the arena/total beam breaks for the interval) × 100, and number of rears were calculated during the first 5 min interval.

### Auditory-cued fear associative learning

Auditory-cued fear associative learning was assessed using the Near-IR Video Fear Conditioning System (MedAssociates, Inc., St. Albans, VT USA) as previously described^13,14^, Briefly, mice were habituated to the testing room 30 min prior to behavioral testing, then placed into the illuminated test chamber and acclimated for 2 min with 68 dB white noise; mice were then exposed to a series of 8 conditioning stimuli consisting of a 6 s tone (3500 Hz, 80 dB done, CS) co-terminating with a 2 s 0.6 mA foot shock (unconditioned stimulus, US), followed by a 30 s interval and was repeated 7 more times for a total of 8 tone intervals. Mice remained in the chamber for 60 s after the final conditioned stimulus/unconditioned stimulus (CS/US) pairing, and then were returned to their home cages. Twenty-four hours after the acquisition trial, mice were returned to the testing room and were re-acclimated for 30 min prior to testing. Hippocampal-dependent contextual fear retention was assessed by returning mice to the unmodified chambers, and freezing behavior was recorded for 180 s. Mice were returned to their home cages after this test. Four hours following the contextual test, mice were re-tested for amygdala-dependent associative memory trial in a modified chamber with the context altered by changing the external and internal environment. The testing room was dimly lit with the addition of white noise from a fan. The chamber system was modified in shape, texture, and odor by the addition of a smooth white plastic semi-circular internal surround with a solid floor, lights were extinguished, and a gauze pad with a drop of vanilla was present outside of the testing chamber to provide a novel olfactory environment. Mice were then tested for retention of the CS/US pairing following the same cue presentation schedule as used for the acquisition trial, without the foot shock administration. Across all testing, duration of freezing and number of freezing bouts (lack of movement with the exception of breathing) was quantified automatically by the VideoFreeze software (VideoFreeze Video Fear Conditioning Software, Med Associates Inc., St. Albans, VT; https://med-associates.com/product/videofreeze-video-fear-conditioning-software/) and is reported here as percent time freezing per component interval.

### Resin perfusions and µCT scanning

Twenty-four to seventy-two hours after completion of behavioral testing, a subset of pseudorandomly selected mice (LD n = 8, CJL n = 4) were euthanized, and perfusions for brain vascular corrosion casting were performed between ZT 5–8 (13:00 to 16:00 h) as previously described^15^. Mice received a lethal dose of sodium pentobarbital, then, after confirming deep anesthesia, the thoracic region was dissected to expose the heart and lungs. Mice were then transcardially perfused at a rate of 4 mL/min with 15 ml of 25U/mL saline heparin solution, immediately followed by 15 mL of 4% paraformaldehyde. During paraformaldehyde perfusion, fresh PU4ii-RO resin (VasQTec) was mixed and degassed according to manufacturer’s guidelines. After paraformaldehyde perfusion , mice were immediately perfused with 20 mL of fresh PU4ii-RO resin. After perfusion, the resin was allowed to harden for 5 days at 4 °C. Heads were dissected and skulls were removed and decalcified using 5% formic acid (BDH4554; VWR International) for 5 h. The brains were then gently dissected, and casts were isolated by dissolving the remaining tissue with 7.5% potassium hydroxide (KOH) (BDH7622; VWR International) in two 12 h washes at 50 °C. Resin casts were rinsed with milli-Q water, and then lyophilized prior to mounting on pedestals for scanning.

Cerebrovascular corrosion casts were imaged using the SkyScan μCT Scanner (1272 Bruker; https://www.bruker.com/en/products-and-solutions/preclinical-imaging/micro-ct/skyscan-1272.html), following 50 kV/200 µA without filter and 360°, 0.17° interval rotation. There were 900 ms exposures with 4 frame averages/step producing a 2.7 µm^3^ isotropic voxel resolution. Images were reconstructed at 15% ring artifact reduction at 3, smoothing at 0, 0.02–0.40 dynamic range images with compensation across individual samples using NRecon (Bruker; https://www.bruker.com/en/products-and-solutions/preclinical-imaging/micro-ct/skyscan-1272.html) the reconstruction software to transition 2D slice images to 3D volume reconstructions, according to manufacturer’s guidelines and previous studies^15^. Volumes were resliced coronally, loaded into CTAn (Bruker; https://www.bruker.com/en/products-and-solutions/diffractometers-and-x-ray-microscopes/3d-x-ray-microscopes/xrm-software.html), a 2D/3D image analysis and processing software. The hippocampus and isocortex were manually segmented between bregma − 1.6 to − 2.6 and were interpolated at 0.1 mm distances. Manual segmentations for boundary and guidelines for specific brain regions of interest were determined using the Allen Institute mouse brain atlas to reference (https://mouse.brain-map.org/static/atlas). Following segmentations, 3D vasculature datasets were analyzed with VesselVio (https://jacobbumgarner.github.io/VesselVio/), an open-source image segmentation and visualization software developed by our lab^16,17^. Using VesselVio, 10 µm filters were applied to isolated segments and 5 µm filters for endpoint segmenting to account for skeletonization errors.

### Statistical analyses

Survival data were assessed using the Mantel-Cox Test. Spontaneous alternation in the Y maze task, and the comparisons between pre-tone (habituation) and post-tone freezing during the acquisition trial and CS/US trial of the auditory-cued fear associative learning task were analyzed using a two-way ANOVA; one LD female was excluded from spontaneous alternation analysis due to a corrupted video file. Other comparisons including body mass, and behavior data including the acquisition trial and the associative memory trial of the auditory-cued fear associative learning task, were analyzed using repeated measures three-way ANOVA for time, sex, and lighting condition. For significant ANOVAs, post-hoc comparisons were made using Fisher’s LSD test. For body mass assessment, only mice that survived throughout the experimental timeline to collection were included. Segmented vasculature hippocampal and isocortex volumes were analyzed using VesselVio^16^ with 10 µm filters for isolated segments and 5 µm filters for endpoint segmenting. Vasculature datasets were collapsed between sexes due to the limited number of casts collected. Two resin-casted vascular models from the LD cohort in the hippocampal dataset were not analyzed due to resin pocket formations in that brain region. Unpaired two-way *t*-tests were performed between vasculature datasets across lighting conditions. Data were tested for normality using the Shapiro–Wilk tests.. Outlier detection was conducted using Grubb’s Outlier Test; defined as a z-score > 2. A maximum of one outlier per treatment group was removed from analysis. Differences were considered statistically significant if p ≤ 0.05. All analyses were conducted using GraphPad Prism 9.

## Results

### Male and female mice do not display increased mortality or changes to body mass following chronic phase advances

Survival and lifespan were assessed across 8 weeks of exposure to stable lighting conditions or consecutive chronic phase advances in aged male and female mice (Fig. 1a). There were no significant differences in survival during this period among male and female mice housed in stable circadian lighting or exposed to chronic phase advances using the Mantel-Cox test (Fig. 1b; p > 0.05). Body mass (reported as a percentage compared to baseline) was assessed weekly across 8 weeks of typical light dark conditions or jet lag using a Three-way ANOVA for time, lighting condition, and sex. All mice lost body mass across the experiment (p < 0.05; Fig. 1c,d). There were no effects of sex (F_1,47_ = 1.653; p > 0.05), chronic phase advances on mass loss (F_1,47_ = 1.582; p > 0.05), or interaction of lighting condition on body mass loss (p > 0.05).

### Chronic phase advances reduce spatial recognition memory in female mice

Spatial working memory was assessed using a Y-maze task after 8 weeks of chronic phase advances by comparing the percentage of correct alternations during free exploration of the maze (Fig. 2a,b). Chronic jet lag reduced percent correct alternations, which is indicative of impaired spatial memory in female mice (p < 0.05). Furthermore, sex interacted with jet lag to alter this outcome in male mice (F_1,46_ = 8.439; p < 0.01; Fig. 2c). Jet lagged females had reduced correct percentages of spontaneous alternations compared to jet-lagged male counterparts (p < 0.05), as well as females housed in stable circadian conditions (multiple comparisons; p < 0.05). There were no sex differences among mice housed under stable circadian conditions (p > 0.05).

### Female mice exposed to chronic phase advances reduce acquisition during auditory-cued fear associative learning task

**Figure 3:**
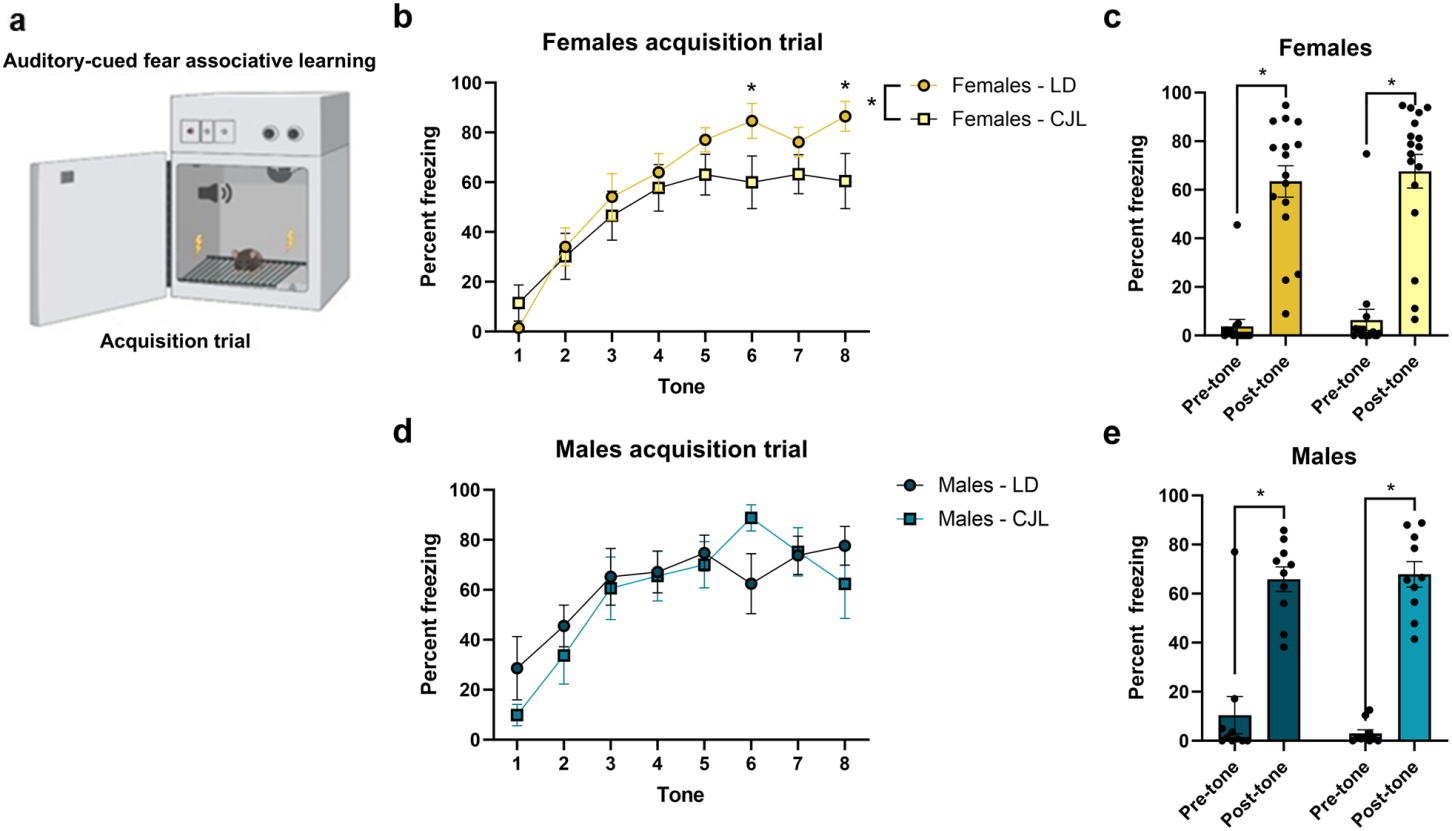
Chronic phase advances reduce fear responses in female mice (**a**) Schematic illustration of behavioral paradigm for the acquisition trial of the auditory-cued fear conditioning test. (**b**) Females exposed to chronic phase advances reduce freezing across tone presentations during auditory-cued fear conditioning acquisition compared to stable lighting conditions (p < 0.05) (**c**) During the acquisition phase, both CJL and LD females significantly increased freezing behavior (%) post-tone presentation compared to pre-tone habituation indicating females acquired the learning task (p < 0.05) (**d**) No differences between stable circadian conditions and males housed under chronic phase advances during the acquisition auditory-cued fear task (p > 0.05). (**e**) CJL and LD males significantly increased freezing behavior post-tone compared to pre-tone (p < 0.05) Data are represented as Mean ± SEM (Male LD n = 10, Male CJL n = 10, Female LD n = 16, Female CJL n = 15) *p < 0.05.

We next assessed spatial and associative memory in an auditory-cued fear conditioning test as an additional measure of cognitive function and memory (Fig. 3a). All mice acquired the association of the tone and the foot shock as evidenced by an increase in freezing during tone presentation across trials (males F _7,126_ = 10.94; females F _7,203_ = 26.93; p < 0.0001; Fig. 3b,d). All mice displayed more freezing behavior after the pairings than before (males F_1,18_ = 115.2; females F_1,58_ = 158.1; p < 0.0001; Fig. 3c,e), chronic phase advances did not affect this measure (p > 0.05). During the acquisition trial, there was a main interaction effect of time, lighting condition, and sex (F_3,392_ = 8.875; p < 0.0001) and tones (F_7,329_ = 33.93; p < 0.0001). Females exposed to chronic phase advances display significantly reduced freezing compared to females housed in stable circadian conditions (post-hoc: Tone 6 p < 0.05, Tone 8 p < 0.005) (Fig. 3b). In males, there was no effect of phase advances on freezing behavior during acquisition (p > 0.05) (Fig. 3b). Percent freezing after acquisition of the CS-US pairings was not affected by sex or chronic phase advances (p > 0.05) (Fig. 3c).

### Sex and phase advances do not alter contextual dependent fear, but jet lagged males display reduced freezing during tone retention trials

**Figure 4:**
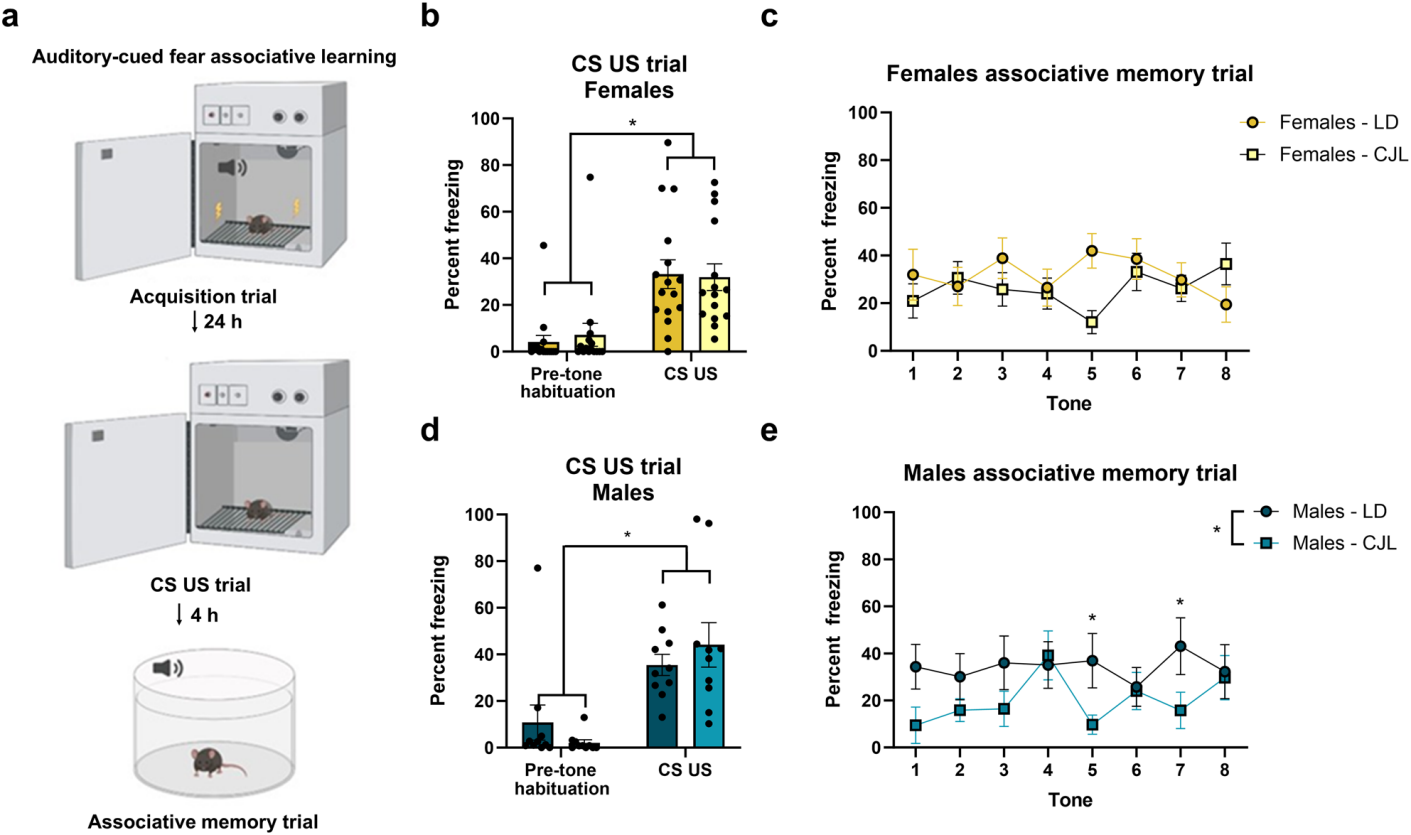
Chronic phase advances reduce retention of conditioned fear responses in male mice (**a**) Schematic illustration of behavioral paradigm for the acquisition, CS US, and associative memory trial of the auditory-cued fear associative learning task. (**b**, **c**) All mice increased freezing to the context compared to pre-trials, suggesting that mice retain fear to context dependent cues (p < 0.05), freezing was not affected by sex or phase advances (p > 0.05). (**d**) No significant differences in the percentage of freezing behavior between lighting conditions in females (**e**) males exposed to chronic phase advances display significantly reduced freezing behavior compared to stable lighting conditions 24 h after acquisition during associative memory retention trials (p < 0.05). Data are represented as Mean ± SEM (Male LD n = 10, Male CJL n = 10, Female LD n = 16, Female CJL n = 15), *p < 0.05.

Twenty-four h after acquisition, mice were placed back into the unmodified fear conditioning chambers to assess contextual fear retention. 4 h after, mice were re-tested for an associative memory trial using a modified chamber environment with altered contextual cues in a new environment (chamber dimension and shape, lights, odor, testing room and apparatus settings) to avoid context-dependent cues or freezing, and were re-assessed using the same acquisition tone trial with the exception of the paired foot shock (Fig. 4a). Both males and females froze more during the retention trials than during pre-CS-US presentation during acquisition, indicating that all mice retained context-dependent memory (p < 0.05) (Fig. 4b,d). In the modified environment, there was an interaction effect between tone and lighting condition (F_7,329_ = 2.707; p < 0.0001). Freezing behavior did not differ due to phase advances during retention tone trials in females (p > 0.05) (Fig. 4c), whereas males exposed to chronic phase advances reduced freezing compared to males housed in stable circadian conditions (Repeated Measures Two-way ANOVA; RMANOVA (F_1,144_ = 9.356; p < 0.005, post-hoc; Tone 5 p < 0.05, Tone 7 p < 0.05; Fig. 4e).

### Exposure to chronic phase advances increases anxiety-like behaviors in male mice

**Figure 5:**
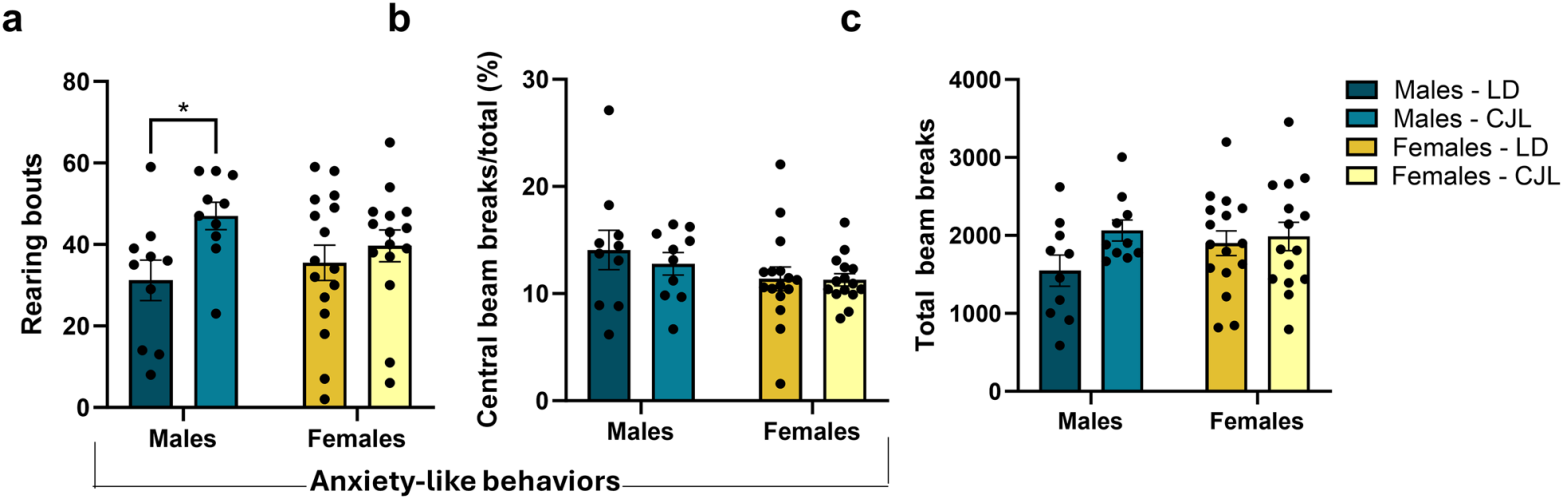
Exposure to chronic phase advances increases anxiety-like behavior in male mice. (**a**) Male mice exposed to chronic phase advances increase rearing in an open field compared to males housed in stable lighting conditions (p < 0.05). (**b**, **c**) there were no differences in central tendency or total locomotor activity due to phase advances or sex. Data are represented as Mean ± SEM (Male LD n = 10, Male CJL n = 10, Female LD n = 16, Female CJL n = 15), *p < 0.05.

To determine whether chronic phase advances resulted in behavioral changes in locomotor activity and anxiety-like behaviors, the mice were assessed using the open-field test. Across the first 5 min of exploring a novel open-field, males exposed to chronic phase advances had significantly elevated number of rears relative to mice housed in stable circadian conditions (Two-way ANOVA F_1,47_ = 5.251; p < 0.05, post-hoc analyses p < 0.05) (Fig. 5a), and there were no differences among females due to circadian phase advances (p > 0.05). Central tendency and total locomotor activity were not significantly different across sex or circadian condition (p > 0.05) Fig. 5b,c).

### Chronic phase advances alter vascular tortuosity in the isocortex, but vasculature in the hippocampus does not change

**Figure 6:**
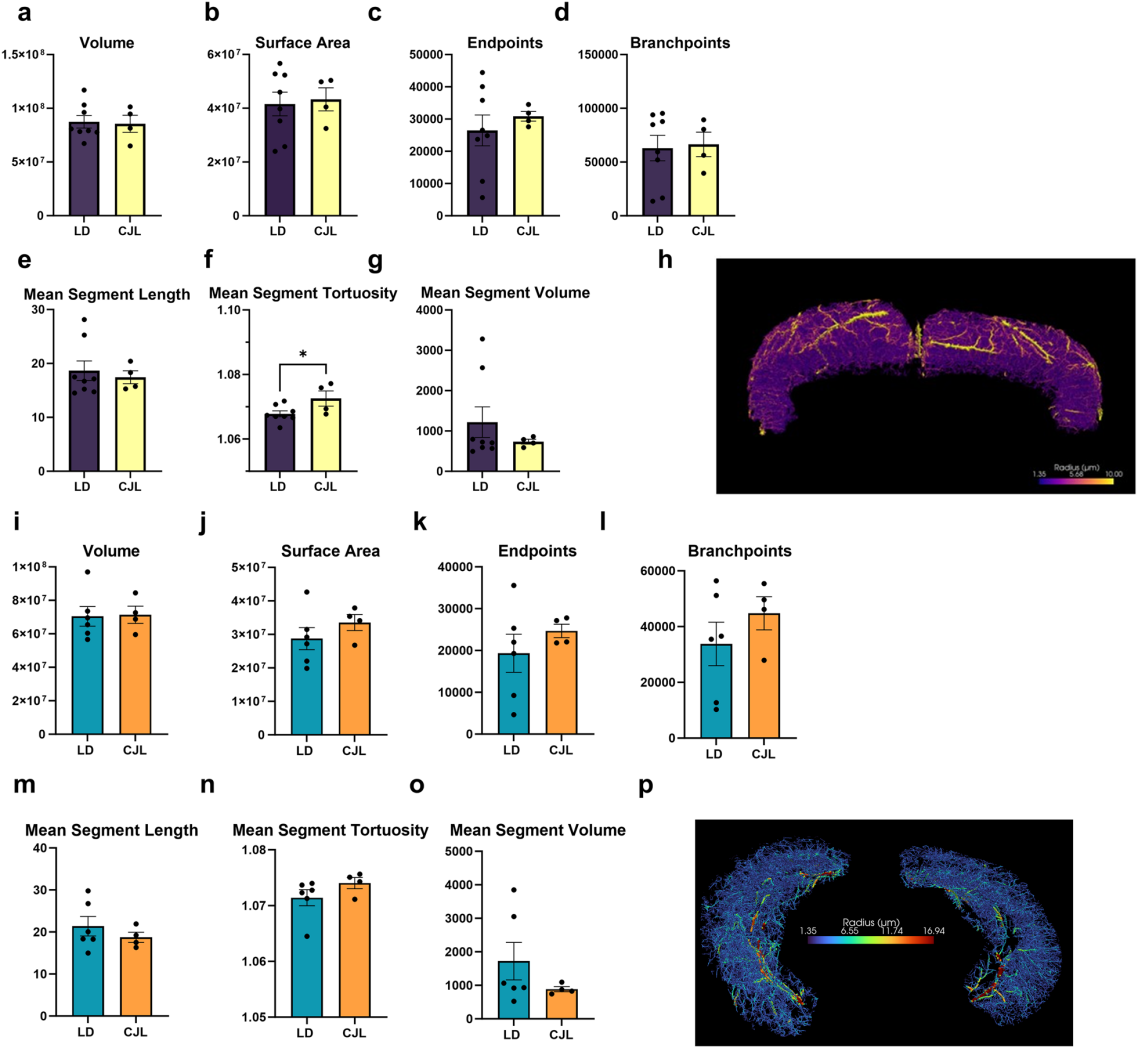
Chronic phase advances increase vessel tortuosity in the isocortex of aged mice. Graphs visualizing vasculature parameters in the isocortex (**a**–**h**) and hippocampus (**i**–**p**) using VesselVio, including 3D segmentations of the isocortex (**h**) and hippocampus (**p**). Aged mice maintained under stable circadian conditions or exposed to chronic phase advances do not display significant differences in vasculature or segment characteristics vascular volume, surface area, branch points, or network length (p > 0.05), however, (**f**) chronic phase advances increase vascular tortuosity compared to stable circadian controls (p < 0.05). There are no significant differences in vascular characteristics observed in the hippocampus (p > 0.05) (**i**–**o**). Data are represented as Mean ± SEM (Hippocampus; LD n = 6, CJL n = 4, Isocortex LD n = 8, CJL n = 4) *p < 0.05. Two resin-casted vascular models from the LD cohort in the hippocampal dataset were not analyzed due to resin pocket formations in that brain region.

To determine whether increased cognitive impairments in aged mice exposed to chronic phase advances were associated with changes in brain vasculature, cerebrovascular casts were assessed in the isocortex and hippocampus. There were no differences in network volume, vascular density, and surface area of the cerebrovascular network in these brain regions due to chronic phase advances (p > 0.05, Fig. 6a–e; isocortex). We next assessed the mean characteristics of individual vessel segments within each region to determine whether components of the vessels comprising vasculature were changed. Exposure to chronic phase advances increased tortuosity in the isocortex (Fig. 6f; F_3,7_ = 3.376; p < 0.05) compared to aged mice housed under stable circadian conditions. No other aspects of segment characteristics were different between lighting conditions in either brain region (Fig. 6a–e,g; isocortex, and Fig. 6i–o; hippocampus; p > 0.05).

## Discussion

Here, we report that chronic phase advances reduce spatial working memory and auditory-cued fear associative learning tasks in aged mice. Our study provides evidence that disruptions to circadian rhythms differentially affect aspects of cognitive function between sexes in aged mice. These results suggest that exposure to chronic phase advances affect both hippocampal dependent spatial working memory in females and the acquisition of auditory-cued fear associative learning tasks. From our results, females exposed to chronic phase advances may experience impairments in the hippocampus, amygdala, and cortical regions involved in fear conditioning, whereas males exposed to chronic jet lag likely only experience impairments in the amygdala. To our knowledge, this is one of the first studies to report a sex difference in these aspects of cognitive function after exposure to chronic jet lag in aged populations. Both basic and clinical studies support the hypothesis that chronic jet lag affects cognitive function. In a human study, female flight attendants exposed to chronic jet lag have increased learning and memory deficits that correspond with atrophy of the temporal lobe compared to flight attendants flying north–south routes^18^. Another study reported elevated levels of cortisol that were associated with reduced temporal lobe volume and impaired spatial learning and memory in flight crews exposed to repeated jet lag compared to short distance travel^19^ Impairments in learning and memory after disrupted circadian rhythms in otherwise healthy adult rodents has also been well characterized. For example, adult female hamsters exposed to phase advances displayed significantly impaired learning and memory associated with reduced neurogenesis and cell proliferation in the hippocampus. Further, these deficits persisted after returning to stable light–dark cycles suggesting potential for long term cognitive consequences^20^. Together, these studies, in common with our results, provide converging evidence that (1) circadian rhythms contribute to cognitive aging and (2) neurodegenerative disease progression differences could be due to differences in physiological progression of cognitive aging or advanced neurodegeneration after circadian disruption in females compared to males. We have previously demonstrated that females exhibit dysregulated immune responses, significantly accelerated aging, and shortened lifespan after 24 weeks of exposure to chronic dim light at night^21^.

Coinciding with increased prevalence of dementia in the aging human population, previous research has characterized the relationship among neurodegeneration, vascular dysfunction, and cognitive decline^22^. In the current study, we observed that exposure to circadian disruption from chronic phase advances increases vascular tortuosity in aged populations compared to control age-matched mice housed in stable circadian conditions. Tortuosity of vasculature results from hemodynamic changes to blood flow resulting in weakening of arterial walls or twisting of vasculature that increases across age^23^, but can be indicative of systemic disease^24^ and is correlated with vascular pathologies in human studies^25^. Other preclinical models characterizing aging, reported a significant reduction in blood velocity and blood volume in the cerebral cortex, along with increases in vascular tortuosity using super-resolution ultrasound localization microscopy (ULM) imaging^26^. Tortuosity can elevate blood flow and pressure^24^ that can weaken arterial walls and reduce axial tension over time^27^ increasing risk of vascular disease and injury. Clinical studies evaluating patients with intracerebral artery (ICA) aneurysms reported increased ICA tortuosity^15^. Other vascular diseases that have been correlated with increased tortuosity of the ICA include patients after subarachnoid hemorrhage^28^. Here, our results suggest that coinciding with cognitive impairment phenotypes observed in our mouse model, we observed increased tortuosity that may contribute to reduced cognitive function, as well as could contribute as a greater risk for vascular disease, including stroke.

The vasculature of young adult rodents has also been altered in experiments that disrupted circadian rhythms via exposure to dim light at night (dLAN); in many cases dLAN reduces angiogenesis and vascular permeability factors such as vascular endothelial growth factor (VEGF)^17,29–31^. Our results are consistent with others across several experimental approaches that disruptions to circadian rhythms changes brain vasculature that likely increases risk for vascular-related diseases and pathologies. Although our current and previous research findings provide supporting evidence that circadian rhythm disruption alters vascular changes, there are several pathologies and risk factors that contribute to the development of VCID. Several other animal models are currently reported in the vascular dementia literature. For example, other groups investigating VCID induce VCID-like phenotypes through bilateral carotid artery stenosis (BCAS) that produce subcortical ischemic injury and hippocampal atrophy^32^, chronic hypoperfusion and genetic or diet-induced hyperhomocysteinemia^33^, spontaneously hypertensive rat models^34^, cerebral autosomal dominant arteriopathy with subcortical infarcts and leukoencephalopathy (CADSIL) mouse model^35^, or pair carotid artery occlusion with modifiable risk factors such as high fat diet to induce metabolic disease^36^. Despite the number of broad pathologies contributing to VCID phenotypes in animal models, sex specific differences in dementia subtypes persist. Chronic cerebral hypoperfused adult (3–7 month old) female mice maintained on a high fat diet (60% fat) displayed higher impairment in spatial learning, metabolic, neuronal pathologies (including astrogliosis and greater hippocampal insoluble amyloid beta 40) compared to group-matched males or sham (carotid artery occlusion surgery only) groups^37^. Our data suggest that, independent of genetic, surgical, or diet-induced manipulations, disruption of circadian rhythms alone in aged rodents is sufficient to alter structural changes in vasculature. Mouse models are an important tool for understanding mechanisms of dysregulated physiological rhythms and circadian disorders, particularly, for aged populations that are more susceptible to changes in physiological function and at greater risk of disease than young animals. Future investigation into the molecular mechanisms underlying VCID should consider circadian components in other VCID animal models. Further, the investigation of why females are more susceptible than males to circadian influences in late life warrants further examination. One limitation of this experiment is that we could not discern sex differences among resin casts due to limitations in the number of animals we casted. Future studies may be able to determine whether changes in tortuosity are associated with alterations to cerebral blood flow, and whether these parameters are different between aged male and female mice.

Taken together, the present study provides evidence supporting a convergence among aging, changes to structural components of vasculature, and cognitive impairments increasing risk for cognitive dementias in aged mice exposed to circadian rhythm disruption from chronic phase advances. Females experiencing chronic phase advances, had increased spatial working memory deficits during a spontaneous alternation task, and significantly reduced freezing behavior during the acquisition trials of an auditory-cued fear associative learning task. Conversely, males exposed to chronic phase advances exhibited reduced retention of auditory-cued fear associative learning 24 h after acquisition and increased anxiety-like behaviors during an open field task compared to mice housed in stable lighting conditions. These cognitive deficits were associated with increased vascular tortuosity in the isocortex, which is associated in humans with increased risk of vascular diseases and changes to cerebral blood flow. In conclusion, these results demonstrate that exposure to chronic phase advances results in sex-dependent differences, by increasing cognitive aging and impairments through altering spatial recognition memory, and auditory-cued fear associative learning in aged mice^12,16^.

## Data Availability

The datasets generated during and/or analyzed during the current study are available as a preview in this published Mendeley Data Repository dataset: Liu, Jennifer (2024), “WVU_JAL_CJLProject2023”, Mendeley Data, V1, 10.17632/8gc9m5y9by.1. Data are available from the corresponding author on reasonable request.

## References

[1] Iadecola C (2019). Vascular cognitive impairment and dementia. J. Am. Coll. Cardiol..

[2] Kalaria RN (2018). The pathology and pathophysiology of vascular dementia. Neuropharmacology.

[3] Hastings MH, Maywood ES, Brancaccio M (2018). Generation of circadian rhythms in the suprachiasmatic nucleus. Nat. Rev. Neurosci..

[4] Zhang F (2020). The effect of jet lag on the human brain: A neuroimaging study. Hum. Brain Mapp..

[5] Bouman EJ (2023). Social jet lag and (changes in) glycemic and metabolic control in people with type 2 diabetes. Obesity.

[6] Roenneberg T (2023). How can social jetlag affect health?. Nat. Rev. Endocrinol..

[7] Khachiyants N, Trinkle D, Son SJ, Kim KY (2011). Sundown syndrome in persons with dementia: An update. Psychiatry Investig..

[8] Kondratova AA, Kondratov RV (2012). The circadian clock and pathology of the ageing brain. Nat. Rev. Neurosci..

[9] Leng Y, Musiek ES, Hu K, Cappuccio FP, Yaffe K (2019). Association between circadian rhythms and neurodegenerative diseases. Lancet Neurol..

[10] Cronin P (2017). Circadian alterations during early stages of Alzheimer’s disease are associated with aberrant cycles of DNA methylation in BMAL1. Alzheimers Dement..

[11] Bellanti F (2017). Alterations of clock gene RNA expression in brain regions of a triple transgenic model of Alzheimer’s disease. J. Alzheimer’s Dis..

[12] Walker WH (2021). Artificial light at night reduces anxiety-like behavior in female mice with exacerbated mammary tumor growth. Cancers.

[13] Cissé YM, Peng J, Nelson RJ (2016). Dim light at night prior to adolescence increases adult anxiety-like behaviors. Chronobiol. Int..

[14] Walton JC, Haim A, Spieldenner JM, Nelson RJ (2012). Photoperiod alters fear responses and basolateral amygdala neuronal spine density in white-footed mice (*Peromyscus leucopus*). Behav. Brain Res..

[15] Labeyrie P-E (2017). Cervical artery tortuosity is associated with intracranial aneurysm. Int. J. Stroke.

[16] Bumgarner JR, Nelson RJ (2022). Open-source analysis and visualization of segmented vasculature datasets with VesselVio. Cell Rep. Methods.

[17] Bumgarner JR (2023). Acute exposure to artificial light at night alters hippocampal vascular structure in mice. Science.

[18] Choy M, Salbu RL (2011). Jet lag: Current and potential therapies. P T.

[19] Cho K (2001). Chronic ‘jet lag’ produces temporal lobe atrophy and spatial cognitive deficits. Nat. Neurosci..

[20] Gibson EM, Wang C, Tjho S, Khattar N, Kriegsfeld LJ (2010). Experimental ‘jet lag’ inhibits adult neurogenesis and produces long-term cognitive deficits in female hamsters. PLoS ONE.

[21] Liu JA (2022). Chronic exposure to dim light at night disrupts cell-mediated immune response and decreases longevity in aged female mice. Chronobiol. Int..

[22] Raz L, Knoefel J, Bhaskar K (2016). The neuropathology and cerebrovascular mechanisms of dementia. J. Cereb. Blood Flow Metab..

[23] Amemiya T, Bhutto IA (2001). Retinal vascular changes and systemic diseases: Corrosion cast demonstration. Ital. J. Anat. Embryol..

[24] Hughes AD (2006). Quantification of topological changes in retinal vascular architecture in essential and malignant hypertension. J. Hypertens..

[25] Chen Y-C (2020). Correlation between internal carotid artery tortuosity and imaging of cerebral small vessel disease. Front. Neurol..

[26] Lowerison MR (2022). Aging-related cerebral microvascular changes visualized using ultrasound localization microscopy in the living mouse. Sci. Rep..

[27] Han H-C (2012). Twisted blood vessels: Symptoms, etiology and biomechanical mechanisms. J. Vasc. Res..

[28] Krzyżewski RM (2022). Subarachnoid hemorrhage from ruptured internal carotid artery aneurysm: Association with arterial tortuosity. World Neurosurg..

[29] Aubrecht TG, Weil ZM, Magalang UJ, Nelson RJ (2013). Dim light at night interacts with intermittent hypoxia to alter cognitive and affective responses. Am. J. Physiol. Regul. Integr. Comp. Physiol..

[30] Chellappa SL, Vujovic N, Williams JS, Scheer FAJL (2019). Impact of circadian disruption on cardiovascular function and disease. Trends Endocrinol. Metab..

[31] Walker WH (2020). Acute exposure to low-level light at night is sufficient to induce neurological changes and depressive-like behavior. Mol. Psychiatry.

[32] Nishio K (2010). A mouse model characterizing features of vascular dementia with hippocampal atrophy. Stroke.

[33] Jadavji NM (2015). Elevated levels of plasma homocysteine, deficiencies in dietary folic acid and uracil–DNA glycosylase impair learning in a mouse model of vascular cognitive impairment. Behav. Brain Res..

[34] Tayebati SK, Tomassoni D, Amenta F (2012). Spontaneously hypertensive rat as a model of vascular brain disorder: Microanatomy, neurochemistry and behavior. J. Neurol. Sci..

[35] Cognat E, Cleophax S, Domenga-Denier V, Joutel A (2014). Early white matter changes in CADASIL: Evidence of segmental intramyelinic oedema in a pre-clinical mouse model. Acta Neuropathol. Commun..

[36] Abi-Ghanem C (2023). Sex differences in the effects of high fat diet on underlying neuropathology in a mouse model of VCID. Biol. Sex Differ..

[37] Gannon OJ (2022). High-fat diet exacerbates cognitive decline in mouse models of Alzheimer’s disease and mixed dementia in a sex-dependent manner. J. Neuroinflamm..

